# Extracellular Vesicles: A Novel Mode of Viral Propagation Exploited by Enveloped and Non-Enveloped Viruses

**DOI:** 10.3390/microorganisms12020274

**Published:** 2024-01-28

**Authors:** Shruti Chatterjee, Ramina Kordbacheh, Jon Sin

**Affiliations:** Department of Biological Sciences, University of Alabama, 1325 Hackberry Lane, Tuscaloosa, AL 35401, USA; schatterjee6@ua.edu (S.C.); rkordbacheh@crimson.ua.edu (R.K.)

**Keywords:** viruses, exosomes, microvesicles, extracellular vesicles

## Abstract

Extracellular vesicles (EVs) are small membrane-enclosed structures that have gained much attention from researchers across varying scientific fields in the past few decades. Cells secrete diverse types of EVs into the extracellular milieu which include exosomes, microvesicles, and apoptotic bodies. These EVs play a crucial role in facilitating intracellular communication via the transport of proteins, lipids, DNA, rRNA, and miRNAs. It is well known that a number of viruses hijack several cellular pathways involved in EV biogenesis to aid in their replication, assembly, and egress. On the other hand, EVs can also trigger host antiviral immune responses by carrying immunomodulatory molecules and viral antigens on their surface. Owing to this intricate relationship between EVs and viruses, intriguing studies have identified various EV-mediated viral infections and interrogated how EVs can alter overall viral spread and longevity. This review provides a comprehensive overview on the EV-virus relationship, and details various modes of EV-mediated viral spread in the context of clinically relevant enveloped and non-enveloped viruses.

## 1. Introduction

Extracellular vesicles (EVs) are a diverse group of cell-derived membrane-enclosed structures that act as potent mediators of intracellular communication [1]. They are secreted by a wide range of cell populations including (but not limited to) epithelial cells, antigen-presenting cells, tumor cells, and immune cells. EVs have also been shown to have significant potential in diagnostic and therapeutic applications [2,3,4,5]. Originally, EVs were referred to as cellular “garbage bags” that assisted cells in ejecting waste products. The field of EV research reached a major milestone in the 1980s, when seminal studies by Rose Johnstone and Philip Stahl’s groups characterized exosomes after they identified them as nano-sized membrane enclosed vesicles shed from developing reticulocytes [6,7,8,9]. Since then, a significant body of research has been conducted on the biogenesis, secretion, and composition of these exosomes [10]. In addition to exosomes, several other distinct categories of EVs have been identified, such as shedding microvesicles and apoptotic bodies. A large group of EVs are known to have significant potential in diagnostic and therapeutic applications, and thus studying these vesicles has piqued curiosity in researchers from varying scientific disciplines.

Several studies suggest that EVs function as unique shuttles that carry lipids, proteins, DNA, RNA, and even miRNAs to adjacent or distant cells during essential biological processes [11,12,13,14]. A wide range of body fluids like blood, saliva, cerebrospinal fluid, breast milk, semen, and urine are known to contain EVs in response to their varying physiological activities [15,16,17,18]. They are known to influence the tumor microenvironment by regulating a multitude of processes such as proliferation, metastasis, angiogenesis, invasion, migration, and drug resistance [19,20]. In addition, they have the ability to cross the blood–brain barrier and allow for coordination with the immune system at a typically immune-privileged site [21,22,23]. Recent findings suggest that EVs have been exploited by a wide range of viruses to egress from the host cell and evade the host immune response [13,24,25]. Due to the cross-talk between pathways involved in EV biogenesis and replication of a number of viruses, EVs can incorporate viral fragments, proteins, viral RNA, or entire virions within themselves and, since EVs exist naturally (even in the absence of viruses), the host immune system often remains unaware of the trafficking of these viral components in a phenomenon which is similar to the Trojan horse tactic [26]. It has been broadly shown that EVs can not only facilitate viral infection but can also suppress it by modulating the host immune response, thereby acting as mediators of intercellular communication between infected and uninfected cells [13,27,28,29,30]. This review summarizes our current understanding on the relationship between EVs and several clinically relevant viruses and highlights both well-established and hypothetical models of viral spread via EVs.

## 2. Extracellular Vesicles: Types, Composition and Biogenesis

Extracellular vesicles (EVs) can exist in a wide range of sizes depending on their biogenesis and composition. However, the consensus classification of EVs includes three major subgroups: exosomes, microvesicles (MVs), and apoptotic bodies [1,31,32]. Several other vesicle subtypes, such as oncosomes and ectosomes, have also been identified, but the exact biogenesis pathway of these subtypes currently remains elusive [1,4,31]. The classification of these vesicles is based on their heterogeneity in size, composition, and the cellular pathways from which they originate [33,34,35].

### 2.1. Exosomes

**Figure 1:**
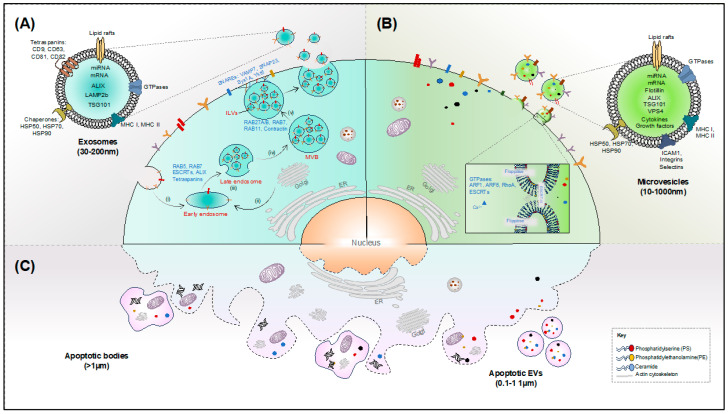
Three major mechanisms of EV biogenesis. (**A**) Release of exosomes into the cell exterior involves three distinct steps: exosome biogenesis, trafficking of multivesicular bodies (MVBs), followed by their fusion with the plasma membrane. Initially, the endosomes are formed by the inward budding of the cell membrane (i) or from the *trans*-Golgi network (ii). These early endosomes then mature to form late endosomes (iii) and finally MVBs (iv). MVBs can finally fuse with the plasma membrane, which leads to the release of exosomes (v). (**B**) Microvesicles are formed due to the direct outward budding of the cell membrane. During microvesicle biogenesis, the enzymes flippase and floppase facilitate lipid remodeling on the outer leaflet of the plasma membrane, resulting in membrane curvature and eventually blebbing of the microvesicle. This process is triggered by cytoskeletal reorganization, increased Ca^2+^ ion concentration, and the action of small GTPases. (**C**) Apoptotic bodies are produced from the cells that undergo apoptosis and contain cell organelles including nuclear fragments.

Exosomes are one of the most studied groups of EVs that are known to play a dual role in disease progression and inhibition [2,36]. They are the smallest of the three main subtypes of EVs, ranging in size from 30 to 200 nm [8,31]. The development of exosomes begins with the formation of an intra-luminal vesicle (ILV) within the lumen of acidic endocytic organelles called multivesicular bodies (MVBs) [1] (Figure 1A). This process can either be regulated by ‘endosomal sorting complex required for transport’ (ESCRT) proteins or ESCRT-independent mechanisms which include membrane lipids and membrane-spanning proteins like tetraspanins [37]. The MVB then fuses with the plasma membrane, with ILVs being subsequently released as exosomes [25]. After being released into the extracellular space, these exosomes interact with the corresponding recipient cells either through direct membrane fusion, micropinocytosis, or via receptor-mediated endocytosis [38]. Following exocytosis, the exosomes still retain several endosome-related proteins, like annexins, lipid raft-associated proteins, and ESCRT accessory proteins, which are further used for intracellular membrane fusion and their transport to the recipient cell [39]. Exosomal membrane proteins include tetraspanins such as CD63 and CD81, fusion proteins like CD9, lysosomal protein LAMP2b, as well as heat shock proteins like HSP70. Among these, tetraspanins are known to play a vital role in mediating exosome formation as well as fusion with the target cells [39,40]. Two additional exosomal proteins include RAB27A, which help in the determination of exosome size, and RAB27B, which are small GTPases, involved in modulating the docking of the MVBs at the plasma membrane during the process of exosome formation [1,38]. The secretion of nucleic acids and proteins via exosomes is critical as it preserves the three-dimensional structure and biological function of the transferred molecules and facilitates their accurate delivery to the target without the need for direct cell–cell contact [41,42]. They are also known to play crucial roles in eliminating redundant proteins and genetic materials (like mRNA and miRNA) from host cells [43], promoting cell-to-cell spread of pathogens like viruses and bacteria, as well as modulating immune-stimulatory and inhibitory processes [1].

### 2.2. Microvesicles

Microvesicles (MVs) are a diverse group of EVs that are formed due to the outward budding of the plasma membrane (Figure 1B). They exist in heterogeneous sizes ranging from 100 nm to 1 μm in diameter and are composed of a repertoire of bioactive components [4,32,44]. The molecular mechanism of MV biogenesis has not been well-characterized; however, their formation involves a complex redistribution of membrane lipids due to the action of several phospholipid transporters like flippase, floppase, and scramblase [45]. Similar to exosomes, ESCRT proteins like ALIX, TSG101, VPS22, and VPS4 are known to be involved in the generation of MVs [46,47]. Apart from that, the small GTPase proteins ARF1, ARF6, and RhoA also facilitate the budding of MVs from the cell membrane of cancer cells [46,48,49,50]. They are formed mostly in lipid-rich microdomains within the plasma membrane and are enriched in proteins such as flotillin-1 and integrins as well as lipids such as phosphatidylserine (PS) on their membrane [51,52]. They can induce acute inflammatory responses in host cells by mediating the transport of pro-inflammatory miRNAs to the targeted cells [45]; this MV-mediated transfer protects the miRNAs from the action of circulating ribonucleases, as well as helps them to repress specific mRNA targets in recipient cells [53]. Moreover, these MVs can also facilitate the rapid release of IL-1β, a pro-inflammatory cytokine that is not secreted via the conventional pathways of protein secretion due to its missing signal sequence [54]. Notably, it has also been shown that the MVs can carry viral contents from the infected cells either within or on their surface following their shedding from the plasma membrane [55,56,57].

### 2.3. Apoptotic Bodies

Apoptotic bodies or apoptosomes are produced from cells undergoing programmed cell death and have a heterogeneous size ranging from 100 nm to 5 µm in diameter. The biogenesis of apoptotic bodies involves several stages including the condensation of nuclear chromatin, subsequent blebbing of the plasma membrane, and distribution of cellular components into distinct membrane-enclosed vesicles (Figure 1C). For years, the role of these apoptotic bodies as EVs has received little attention owing to their large sizes, complex contents, and ability to induce apoptosis. Their easy clearance by phagocytes has raised further questions on their use as drug delivery agents [58]. However, they do play an important role in suppressing inflammation, regenerating tissues, and maintaining tissue homeostasis [59,60,61,62]. Furthermore, like healthy cells, these apoptotic cells also release EVs which are more physiologically similar to exosomes and microvesicles rather than bona fide apoptotic bodies. These EVs are known as ApoEVs and have been thought to have distinct biological functions [63]. However, because this occurs during apoptosis, and because ApoEVs have been shown to have a much wider size distribution than “healthy” EVs, parsing apart ApoEVs from apoptotic bodies remains a challenge.

## 3. Cellular Uptake of EVs

EVs of all three of the aforementioned subtypes can be taken up by recipient cells via two well-known and widely accepted models: cargo delivery by direct membrane fusion and receptor-mediated endocytosis [32,46,64]. The mechanism of direct fusion with the plasma membrane can result in the transport of functional molecules like RNAs into the recipient cells [65,66,67]. However, the majority of experimental evidence suggests that EVs are predominantly taken up via the process of receptor-mediated endocytosis [67,68,69]. Endocytic uptake mechanisms include clathrin-dependent endocytosis, as well as clathrin-independent processes involving caveolins and lipid rafts, phagocytosis, and micropinocytosis [70,71]. All these mechanisms are primarily influenced by a wide range of proteins and glycoproteins which are expressed on the surface of EVs and in the recipient cells [66]. EVs can bind to the target cells by interacting with various receptors such as lectins, heparan sulfate proteoglycans, connexins, and integrins [72,73,74]. Moreover, they can also be internalized and directed to the endosomal pathway where they fuse with lysosomes, thus resulting in the degradation and subsequent recycling of vesicular contents [75].

## 4. Role of EVs in Viral Spread

**Figure 2:**
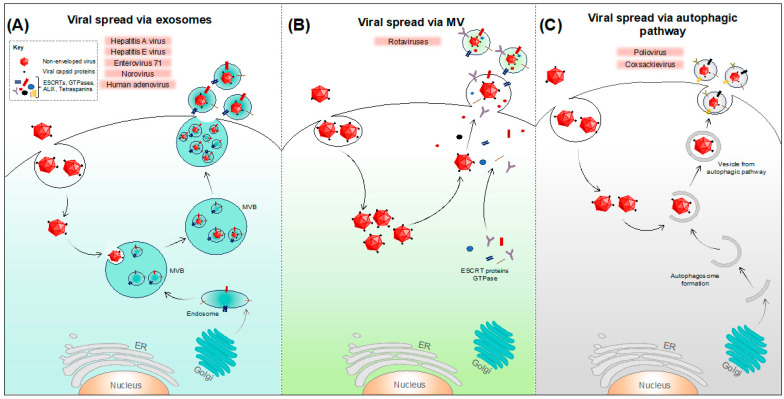
Spread of non-enveloped viruses via extracellular vesicles. Infected cells are known to spread quasi-enveloped virions, viral components (like DNAs, RNAs, miRNAs, proteins), and virus-modified host factors via EVs using different pathways. (**A**) For several non-enveloped viruses like HAV, HEV, EV71, norovirus, and HAdV, virions and other viral factors can be packaged into the ILVs, which will eventually be released into the extracellular milieu after the fusion of MVB with the plasma membrane. (**B**) Virions and viral components of rotaviruses are released from cells being enclosed in shedding microvesicles. (**C**) Some viruses like poliovirus and coxsackievirus can be packaged into vesicles which are derived from the autophagic pathway. These vesicles can either be released by membrane shedding or by membrane fusion (as shown in the schematic), giving rise to quasi-enveloped virions.

**Figure 3:**
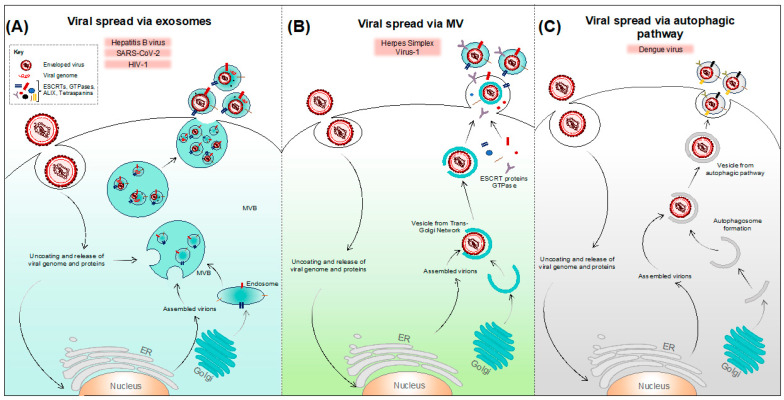
Spread of enveloped viruses via extracellular vesicles. Infected cells are known to spread enveloped virions, viral components (like DNAs, RNAs, miRNAs, proteins), and virus-modified host factors via EVs using different pathways. (**A**) For several enveloped viruses like HBV, SARS-CoV-2, and HIV-1, virions and other viral factors can be packaged into the ILVs, which will eventually be released into the extracellular milieu after the fusion of MVB with the plasma membrane. (**B**) Some viruses like herpes simplex virus-1 are enveloped by the endosomes secreted from the *trans*-Golgi network and are released as enveloped virions, within shedding MVs. (**C**) Viruses like dengue virus can be packaged into vesicles which are derived from the autophagic pathway.

Both enveloped and non-enveloped viruses are known to exploit EVs in order to enter host cells and evade the host immune responses. In addition to transporting infective viral genomes into the target cells, EVs are capable of secreting viral components or complete viral particles to the extracellular space [31,64]. Following viral infection, the EV-mediated release of virions and virus-induced host factors takes place using diverse molecular mechanisms (Figure 2 and Figure 3). Some viruses (like hepatitis A, hepatitis E, hepatitis B, enterovirus 71, human adenovirus, SARS-CoV-2, and HIV-1) are known to exploit multivesicular bodies (MVBs) (Figure 2A and Figure 3A) for the release of virions or viral components into the extracellular medium as exosomes, while others (like rotaviruses, human herpesvirus 1) are known to exploit shedding microvesicles as a mode of viral egress (Figure 2B and Figure 3B). Furthermore, infected cells can also release EV-enclosed viruses via autophagic pathways (as in case of poliovirus, coxsackievirus, and dengue virus), which entails the wrapping of viruses into autophagosomal membranes (Figure 2C and Figure 3C). All these pathways lead to the successful release of virus-laden extracellular vesicles and require the concerted function of various molecular machinery such as the ESCRT complex, tetraspanin-enriched microdomains, and lipid rafts [32]. Furthermore, due to the litany of cellular pathways that are subverted during viral infection, many of these EV-based egress mechanisms may involve altered/aberrant forms of homeostatic EV pathways.

## 5. EVs and Naked Viruses

Recent studies in the field of non-enveloped or ‘naked’ viruses have drawn significant attention as they are now known to exploit EVs in order to facilitate non-lytic cell-to-cell spread and evade the host immune system. As such, released viral particles are no longer naked, but instead enclosed in vesicles which serve as a “quasi-envelope” [64]. These EVs can act in diverse ways once they enter the recipient cells. Apart from being directly infective, they can enhance susceptibility of the neighboring cells to viral infection by inhibiting cellular antiviral responses [76]. Conversely, sometimes they can trigger antiviral responses and cytokine secretion in uninfected cells near the site of infection, thus protecting these neighboring cells from further infection [64].

### 5.1. Hepatitis A Virus

Hepatitis A virus (HAV) is a positive-sense RNA virus that is responsible for 0.5% of deaths worldwide due to viral hepatitis and acute liver failure [77]. Though HAV is classified as non-enveloped, Stanley Lemon et al. demonstrated that the virus can hijack exosome biogenesis pathways to release membrane-cloaked quasi-enveloped Hepatitis A virions (eHAV) [78]. The VP2 and VP1pX capsid domains of the naked virion interact with several membrane components including ALIX, ESCRT-III, and VPS4 from the host ESCRT machinery to be internalized into multivesicular bodies (MVBs) or to bud out through the plasma membrane. Notably, in eHAV particles, the VP1pX protein is not cleaved and processed to the VP1 form. The pX domain of the uncleaved VP1pX protein contains lysine residues which are ubiquitinated to shed out the viral cargo via MVBs and the ESCRT pathway [78,79,80,81]. This, in turn, allows for the subsequent release of eHAV particles bound in a single lipid bilayer. Not only do eHAV membranes protect the virus from proteolytic activity, but circulating eHAV particles are also thought to be protected from host neutralizing antibodies [78,82].

### 5.2. Hepatitis E Virus

Hepatitis E virus (HEV) is a positive-sense RNA virus that is estimated to cause acute liver failure in 0.5–4% of infected individuals, and is particularly prevalent among pregnant women or people with underlying liver diseases [83]. Although it has been proposed that cellular ESCRT machinery is necessary for HEV exit, the origins of HEV membranes are poorly understood [84]. It has been reported that HEV particles could potentially acquire a membrane from MVBs prior to their exit from the cell [84]. It has been reported by Stanley Lemon et al. that during infection, quasi-enveloped HEV (eHEV) is the dominant HEV form that circulates in the blood, whereas naked HEV is more prevalent in the feces [79]. In contrast to naked HEV, eHEV particles shed from the host cell possess the phosphoprotein ORF3, which facilitates their egress from the infected cell [79,85]. ORF3 is only present in serum-derived eHEV particles and plays a pivotal role in masking viral antigens from the action of neutralizing host antibodies [79].

### 5.3. Enterovirus 71

Enterovirus 71 (EV71) is a positive-sense RNA virus and a leading cause of hand, foot, and mouth disease (HFMD), especially in children under the age of five [86,87]. EV71 is also known to affect the CNS and can result in significant and even fatal neurological development such as brainstem encephalitis [88,89]. Similar to HAV and HEV, EV71 is also known to exploit exosomes to infect human intestinal epithelial cells [90] as well as neuronal cells [91]. Zhiwei Wu et al. have suggested that the viral release via exosomes facilitates the transfer of viral RNA and miRNA-146a into neighboring host cells [92]. Exosomal miRNA-146a is known to promote viral replication by inhibiting type 1 interferon responses within the target cell. This preferential packaging of miRNA-146a in exosomes plays a crucial role in suppressing host innate immune responses and eventually promoting non-lytic transmission of EV71 virions [92]. The protein–protein interaction which leads to the aforementioned exosomal release of EV71 involves the interaction of EV71 3A protein with VPS25, which is one of the key proteins involved in the ESCRT-II pathway [93]. In addition to HFMD, EV71 is known to infect the central nervous system (CNS), resulting in severe neurological complications like aseptic meningitis, brain encephalitis, delayed neuronal development, neurogenic pulmonary edema, and reduced cognitive development, as observed in a study by Lingxiang Mao et al. [94]. EV71 is known to utilize small extracellular vesicles (sEVs) to cross the blood–brain barrier (BBB) and eventually infect CNS [95]. Since these sEVs are bound in lipid bilayers, EV71 within these vesicles can successfully breach the BBB, thus circumventing the immune surveillance of the host [94,95].

### 5.4. Norovirus

Norovirus (NoV) is a small single-stranded RNA virus that has a wide range of mammalian hosts, including humans. NoV infection can lead to acute gastroenteritis which results in 70,000 to 200,000 human deaths per year worldwide [96]. In 2018, Marianita Santiana et al. showed the non-lytic dissemination of NoV, which, in turn, promotes viral shedding prior to destruction of the host cell. Not only has it been evidenced that NoV virions are released in EVs, but negative stain electron micrographs also reveal that these EVs have a similar size range as exosomes, and protein analyses show that these particles are enriched with classical exosomes markers such as CD63, CD9, and CD81 [97]. Additionally, the EV membrane can promote persistent infection due to its ability to protect the vesicle-cloaked viral components from host proteolytic activity and recognition by gut mucosal antibodies [97].

### 5.5. Human Adenovirus

The human adenovirus (HAdV) is a double-stranded DNA virus that causes gastrointestinal illnesses like diarrhea, febrile respiratory diseases, and pharyngoconjunctival fever [98,99,100,101]. Exosomes, among other types of EVs, have been connected to the pathogenesis of HAdV infection [102]. It has been demonstrated by Brian Sims et al. as well as Larissa Balakireva et al. that the HAdV capsid protein hexon interacts with an anionic phospholipid dipalmitoyl phosphatidylcholine (DPPC) on the exosome membrane to facilitate its non-lytic spread in alveolar epithelial cells [103,104]. Interestingly, DPPC is known to be one of the major constituents in pulmonary surfactant. Hence, its constant presence in the alveolus brings HAdV in contact with the alveolar epithelial cells, resulting in infection even in the absence of primary or secondary receptors. Moreover, these exosomes are also known to possess T-cell immunoglobulin mucin protein (TIM) on their surface which binds to phosphatidylserine on the recipient cells, thus facilitating their entry even in CAR-deficient cells [100,105,106].

### 5.6. Rotaviruses

Rotaviruses are double-stranded RNA viruses which are responsible for about 20–30% of severe diarrhea cases, especially in children younger than 5 years old [107]. Rotavirus particles are known to be present both in exosomes and microvesicles. By analyzing EVs shed from rotavirus-infected Caco2 and MA104 cells via negative stain electron microscopy, Pavel Iša et al. identified rotavirus virions of widely varying sizes in EVs [108]. Via the visualization of virions carried by EVs in the host’s serum, it is suggested that circulating virus-laden EVs can broadly spread to a number of internal organs such as the intestine, spleen, liver, kidney, lungs, and heart. Interestingly, the majority of viral particles associated with exosomes are recruited to the external surface, whereas in microvesicles viral particles are detected internally. Further studies by Reza Rahbarghazi et al. showed that the viral particles which are present inside the microvesicles are protected from the action of potent host neutralizing antibodies, thus promoting their non-lytic spread [109]. The rotavirus spike protein VP4 has been observed to associate with lipid raft microdomains to facilitate the entry of viral components into both exosomes and microvesicles [108,109]. However, further studies need to be conducted to elucidate the exact mechanism of these interactions.

### 5.7. Poliovirus

Polioviruses (PV) are positive-sense RNA viruses that can cause a number of human diseases, most notably poliomyelitis via infection of the central nervous system (CNS) [110]. For many decades, PV has been used as a model system for studying infections by non-enveloped viruses [111]. Via secretion of virus-laden EVs, PV was shown to be able to mediate non-lytic viral egress [78]. Nihal Altan-Bonne et al. proposed that a secretory autophagic pathway mediates the formation of EV-enclosed PV. A cluster of mature infectious PV particles are wrapped in ER-derived double-membraned organelles which resemble autophagosomes [112]. These non-conventional autophagosomes are rich in PS lipids on both the inner and outer leaflets of their membrane. Contrary to the classical autophagosomes which result in degradation of their cytoplasmic components upon fusing with lysosomes, these virus-laden autophagosomes fuse with the plasma membrane to release PV-laden vesicles into the extracellular medium. The single-membrane PS lipid-enriched vesicles (of size range 200–400 nm) facilitate the collective transfer of multiple viral genomes into the recipient cell for a new round of infection [112]. It has been observed that this cell-to-cell en bloc transmission is more effective in spreading viral infection as well as enhancing viral replication. Additionally, as observed by Esther Bullitt et al., this also enables genetic complementarity between viral quasi species, which improves the replication efficiency of viral genomes that would otherwise be attenuated, thus maintaining their existence in the genetic pool [112,113].

### 5.8. Coxsackievirus B

Coxsackievirus B (CVB) is a positive-sense RNA virus and a common enterovirus which displays multiorgan tropism and leads to severe diseases such as meningitis, pancreatitis, and myocarditis [114,115]. Like PV, CVB is thought to gain access into autophagosomes and exit the host through EVs derived from autophagosomal membranes [56,114]. Roberta A. Gottlieb et al. has shown that following infection, CVB has been seen to localize to mitochondrial networks, induce DRP1-mediated mitochondrial fission, and trigger activation of mitophagy, which may serve as a mechanism of viral entry into autophagosomes destined for release [114]. Furthermore, CVB-induced EVs are enriched with specific miRNAs that are not detected in EVs shed from healthy cells. This has been shown by Jon Sin et al., as they reported that EVs shed from CVB-infected cells were enriched with miR-590-5p, which enhanced downstream CVB infection [116]. Recently, Sidong Xiong et al. showed that the coxsackievirus receptor CAR and coreceptor DAF are enriched on the surface of CVB3-induced EVs, and it is assumed that the presence of these receptors may also be involved in facilitating CVB3 entry to EVs [117].

## 6. EVs and Enveloped Viruses

The envelopes of several viruses share significant similarity with EVs in terms of their size, density, morphology, and protein and lipid composition [80,118]. In some other cases, entire enveloped virions can be detected within EVs, which ultimately results in multiple lipid membranes enclosing the capsid/nucleocapsid. Though it would seem unusual for an already enveloped virus to hijack additional membranes, several advantages have been attributed to these hijacked EVs, such as immune evasion, expanded tropic range, and transmission of multiple virions within a single infectious unit.

### 6.1. Hepatitis B Virus

Hepatitis B virus (HBV) is a reverse-transcribing DNA virus that affects more than 250 million chronic carriers and accounts for nearly 900,000 deaths per year [119]. It has been demonstrated that MVBs are essential for HBV development and egress [120,121,122]. Although HBV is an enveloped DNA virus, Reinhild Prange et al. observed that the virus utilizes host MVB machinery to exit the cell. Several cellular factors such as ubiquitin-interacting adaptor γ2-adaptin and ubiquitin ligase Nedd4 play crucial roles in modulating the late stages of viral assembly and eventual EV-based egress [121]. γ2-adaptin and Nedd4 interact with the HBV core and L envelope proteins which likely regulate the transport of the viral components through the late endosomal pathway [123,124]. They observed that mutation of host ESCRT-III and Vps4 impaired the maturation of HBV, thus forming a detergent-resistant, endosome-derived vesicle [121]. Moreover, S and L envelope proteins also play a novel role in the fusion of endosomal vacuoles to MVBs, thus further establishing the role of MVBs in the HBV release [120,121]. Michinori Kohara et al. further suggested that entire virions and/or viral genomes may be shed in EVs, which helps protect viral particles from host-neutralizing antibodies due to an absence of Hepatitis B surface antigen on the outside of the EV [125].

### 6.2. Severe Acute Respiratory Syndrome Coronavirus-2

Severe acute respiratory syndrome coronavirus-2 (SARS-CoV-2) is a positive-sense RNA virus that causes Coronavirus disease 2019 [126]. Though SARS-CoV-2 has primarily been implicated in respiratory illnesses, it has been shown to affect a multitude of organs including the heart and the brain [126,127]. Furthermore, older individuals with underlying diseases including hypertension, atherosclerosis, or atrial fibrillation are more likely to experience COVID-19-induced stroke [128]. Zhaobing Gao et al. showed that SARS-CoV-2 induces the release of EVs containing numerous live virions [129]. The viral envelope 2-E protein appears to be integral for the release of these vesicles, as transfection with 2-E alone induces plasma membrane blebbing and the eventual release of EVs following the initiation of pyroptosis. Further interrogation revealed that these EVs appear to be comprised of Golgi membranes which colocalize with 2-E [129]. These particles not only allow for neutralizing antibody evasion (thus making vaccination more difficult), but have been suggested to allow for receptor-independent infection, potentially allowing for broadened tropism [129].

### 6.3. Human Immunodeficiency Virus Type 1

The human immunodeficiency virus type 1 (HIV-1) is a retrovirus with a single-stranded RNA genome and is the main cause of acquired immunodeficiency syndrome [130]. Interestingly, HIV-1 particles and exosomes possess many common attributes, which somewhat blurs the lines between the two [131]. Marcos V. S. Dias et al. hypothesized that HIV-1 hijacks common exosome-related tetraspanins such as CD63 and CD81 during budding [131]. Additionally, Detlef Schlöndorff et al. also showed that ESCRT proteins are involved in HIV-1 envelope acquisition and egress, further suggesting overlaps with exosome biogenesis pathways. Infected cells have been reported to induce the release of non-infectious EVs as well, which can either promote or inhibit subsequent infection. For example, EVs released from HIV-1-infected cells have been shown to contain the HIV-1 coreceptor CCR5, and it is thought that this may enhance the viral susceptibility of recipient cells [132]. In contrast, Waldemar Popik et al. had shown that HIV-1-induced EVs contain cellular cytidine deaminase APOBEC3G, which is one of the primary host defense elements that inhibits reverse transcriptase activity; thus, the transfer of APOBEC3G via EVs may propagate antiviral effects against downstream HIV-1 infection [133]. However, the overall in vivo efficacy of this potential antiviral strategy remains unclear, as HIV-1 infection is known to reduce APOBEC3G in activated CD4^+^ T cells, thus potentially limiting the amount that can be trafficked into EVs [133].

### 6.4. Herpes Simplex Virus 1

One of the neurotropic alpha-herpesviruses that can develop slowness in neurons is the common human virus herpes simplex virus 1 (HSV-1), which is a double-stranded DNA virus [134]. Via electron microscopy, Raquel Bello-Morales et al. showed that HSV-1 can disseminate via shedding MVs from infected oligodendrocytes among other cell types, but the exact mechanism is still unknown [135]. These particles were revealed to be enriched with exosomal markers such as CD63 and CD81, as well as autophagosome marker LC3-II. Not only were these HSV-1 MVs shown to be infectious, they were also shown to be able to induce infection in normally resistant Chinese hamster ovarian cells, suggesting that MV-based HSV-1 egress could potentially expand the tropic range. Additionally, it was revealed that treatment with overwhelming amounts of anti-HSV-1 antibody could reduce infection but not entirely; thus, MV membranes may at least partially protect HSV-1 from antibody neutralization [135].

### 6.5. Dengue Virus

Dengue virus (DENV) is a single-stranded positive-sense RNA virus which causes dengue fever and has been recently identified as the second most common cause of acute febrile illness in travelers in every region [136,137]. A study by Hameeda Sultana et al. showed that EVs obtained from DENV-infected cells include viral RNAs and proteins such as DENV-enveloped protein (protein E), which bind to the host cell receptor, tetraspanin domain-containing glycoprotein (Tsp29Fb), and facilitates entry into the host cells. These EVs have been shown to contain the entire DENV genome. Due to its positive polarity, even if intact virions are absent, viral proteins can be immediately translated to initiate viral replication [138]. In addition to this, the presence of autophagy proteins such as LC3-II within infectious DENV EVs has also been reported by Yea-Lih Lin et al., and it was shown that silencing autophagy-related genes, *ATG5/Atg7* in Huh 7 cells, and mouse embryonic fibroblasts resulted in a significant reduction in DENV infection [139]. Moreover, knockdown of ATG9 also limited DENV infection, supporting the notion that DENV utilizes autophagy pathways for viral transmission. As with many other viral EVs described in this review, EV-bound DENV was demonstrated to be resistant to antibody neutralization, thus allowing for enhanced immune evasion [139].

## 7. EVs as Therapeutic Resources

EVs are known to have promising potential in drug or vaccine delivery against several viral infections [140,141,142]. Owing to the presence of a lipid bilayer, they are capable of protecting proteins and nucleic acids from the action of trypsin or nuclease digestion, while delivering their cargo to distant recipient cells. Moreover, these EVs can present self-antigens, as well as activate and modulate host immune responses [143,144,145]. EVs, particularly exosomes, derived from mesenchymal stem cells (MSCs) have been exploited for the treatment of several virally mediated diseases with limited therapeutic options due to their immunomodulatory, anti-inflammatory, and pro-angiogenic properties [141,146,147]. Exosomes are known to retain their membrane integrity during long-term storage and repeated freeze–thawing processes, thus making them physiologically more robust than cells [148]. A pioneering work by Klaus Überla et al. showed that exosomes derived from dendritic cells can be explored as a novel vaccine approach against SARS-CoV infection [141]. They produced a chimeric protein (S^GTM^) by replacing the transmembrane and cytoplasmic domain of the spike S protein of SARS-CoV with the G protein of vesicular stomatitis virus. This chimeric S^GTM^ was efficiently incorporated into exosomes, which were subsequently investigated for immunogenicity in mouse models as a potential vaccine against the deadly virus [141]. Another advantage of these stem cell-derived exosomes is the ability to fine-tune their cargos with specific mRNAs, miRNAs, and proteins. This was demonstrated by Huaichang Sun et al. for porcine reproductive and respiratory syndrome virus (PRRSV), wherein artificial microRNAs (amiRNas) were inserted within exosomes in order to suppress the transcription and translation of PRRSV-specific receptor CD163/sialoadhesin (Sn) in porcine alveolar macrophages [142]. This resulted in a reduction in viral titers, suggesting the significance of these EVs as potent small RNA transfer vehicles in pig cells [142]. Thus, in order to employ these EVs as potential therapeutic resources for viral infections, a thorough understanding of EV structure, biochemistry, and the mechanism in which they modulate viral spread and entry into recipient cells is extremely important.

## 8. Discussion

EVs play diverse roles in viral infection, replication, spread, and pathology. Both enveloped and non-enveloped viruses are known to utilize EVs for their non-lytic dissemination among host cells. In this review, we summarize the EV-mediated cell-to-cell spread of a multitude of clinically relevant viruses. Non-enveloped viruses such as HAV, HEV, EV71, NoV, and HAdV, as well as enveloped viruses such as HBV, SARS-CoV-2, and HIV-1, are known to use exosome secretion pathways for their cellular spread [78,84,90,97,102,120,129,131]. Not only can these EVs trigger antiviral responses in uninfected cells, but they can also protect the enclosed viral particles from the action of host-neutralizing antibodies. In addition to exosomes, microvesicles were also shown to be exploited by rotaviruses (non-enveloped) and HSV-1 (enveloped) [108,135]. For viruses such as CVB, PV, and DENV, EV-mediated spread takes place via secretory autophagic pathways. Unlike traditional autophagosomes, these virus-laden autophagosomes fuse with the plasma membrane to release infected vesicles into the extracellular compartment [56,78,139]. This enables the efficient transfer of viral components or entire virions into the recipient cell for a new round of infection. In certain instances, such as with rotaviruses, cellular dissemination takes place by utilizing a combination of exosome and microvesicle biogenesis pathways [108]. Furthermore, human adenoviruses can manipulate EVs to establish a favorable niche even in cells lacking their primary viral receptors [100,103]. While this review mostly focuses on a selection of clinically important viruses that spread via EVs, it is likely that a myriad of other viruses utilize EVs for cellular spread, which will hopefully be elucidated in future studies. In addition to carrying viral particles, virally induced EVs can distribute viral miRNAs to non-susceptible neighboring cells, thus priming them for infection and, eventually, maintaining viral persistence within the host. This has been observed with hepatitis C virus (HCV), porcine reproductive and respiratory syndrome virus (PRRSV), as well as Kaposi’s sarcoma-associated herpesvirus (KSHV) [28,76,149,150,151,152,153,154,155]. An in-depth understanding of EVs and their role in host–viral interactions is essential for future applications in diagnostics and antiviral therapy.

In recent years, there has been a greater emphasis on studying EV biogenesis and characterization as a means of viral transmission. This is mainly due to the development of several newly emerging techniques to detect, isolate, and analyze viral EVs. Due to the similarity in size and density of EVs and viruses, the isolation of EVs from infectious samples has been a challenging task to date [150,156,157]. Numerous studies have used well-known gold-standard techniques to isolate EVs from infected cells or tissues. Among these, one of the pioneering techniques includes ultracentrifugation, such as differential ultracentrifugation and density gradient ultracentrifugation [158]. Although these are reliable and conventional methods for purifying EVs, there are certain limitations to their use. EV isolation with these techniques leads to non-uniform size distribution and sometimes lysis of EVs [159,160]. Moreover, a chance of co-precipitation of EVs along with other similarly weighted macromolecules like protein aggregates or low-density lipoproteins (LDLs) can occur [161]. These factors have led to the discovery of precipitation-based isolation techniques such as polyethylene glycol (PEG) precipitation and lectin-induced agglutination [162]. Unlike ultracentrifugation, these techniques present a more accessible form of EV isolation from large volumes with centrifugation at comparatively lower speeds [163]. However, they still suffer from some pitfalls, which include challenges inb removing PEG polymers from EVs, slight loss in biological activity of the purified EVs, and co-precipitation of protein aggregates [149,163,164,165]. Recently, more refined EV isolation techniques have been discovered, which allow for increased resolution and discrimination of EV populations. These include size-based isolations such as ultrafiltration, hydrostatic filtration dialysis, size exclusion chromatography, microfluidic-based isolation, and immuno-affinity capture-based isolation [166,167]. EVs can also be separated from free viruses based on their differential thermal stabilities followed by standard precipitation techniques. This has been exhibited by Alexander Khromykh et al. while isolating EVs from West Nile virus (WNV)-infected cells [168]. Moreover, pure EVs can also be isolated using a combination of nanoscale flow cytometry and size exclusion chromatography, with individual EVs being separated from a mixture of viruses and EVs using antigen-specific fluorophores [150]. All these approaches have benefits and caveats depending on their complexity, yield, purity, time allotment, and expense. However, choosing the most suitable technique for EV isolation, processing, and analysis poses one of the major challenges in EV research. Although EVs are known to be physiologically more stable than cells, long-term storage, even in −20 °C or −80 °C, often impacts the functional properties of EVs [169,170,171,172]. This has been observed in a study by Rienk Nieuwland et al., where a significant alteration was observed in membrane phospholipids in EVs after being subjected to just a single freeze–thaw cycle [169]. To date, there have been limited transcriptomic, proteomic, or lipidomic studies that focus on the change in EV cargos mediated by long-term storage [171,173,174]. Moreover, culture conditions like cell passage, cell number, and density can also impact the quality, yield, composition, and bioactivity of EVs [175]. These aspects may vary between exosomes, MVs, and apoptotic bodies due to their structural disparity, and this should be considered when handling and storing general, mixed EV samples.

Despite posing experimental challenges, the study of EVs has shown enormous potential in addressing vital questions in healthcare. Tony Hu et al. have shown that analyzing plasma-derived EVs from COVID-19 patients is beneficial for detecting SARS-CoV-2 infection as early as one day post-infection [176]. EV-incorporated viral genomic RNAs are less likely to contain degraded or off-target RNA, thus making them more beneficial than analyzing total plasma RNA [176]. However, further studies are underway to analyze the functional significance of this detection technique and utilize them as a potent indicator of an active SARS-CoV-2 infection. Moreover, Gale W. Newman et al. showed the presence of HIV-1 protein Nef in EVs derived from urine samples of HIV-1 infected patients [177]. The detection of HIV proteins in these EVs serves as an efficient noninvasive diagnostic tool to monitor and eventually treat the disease [177]. In addition to HIV-1 and SARS-CoV-2 infection, Ariel E. Feldstein et al. demonstrated increased levels of miRNAs such as let7, miRNA-29a, and miRNA-340 in blood EVs from alcoholic liver disease (ALD) patients, thus positioning them as promising diagnostic biomarkers in alcoholic hepatitis patients [178].

The ever-evolving field of EV research needs thorough evaluation in clinically relevant systems [179]. In regard to using EVs as potential antiviral therapeutics, some of the important questions which need to be addressed include refining and uniformly adopting EV isolation techniques, evaluating the efficacy of stoichiometrically modified EVs in affecting viral spread, purifying EVs with specific viral surface antigens for cell or organ-specific delivery, and designing EVs that will block newly evolving viral strains as in the case of SARS-CoV-2. Addressing these questions will help us to fully comprehend the role of EVs in modulating viral spread, potentially allowing for novel therapeutic strategies to combat serious health threats. The major goal of this review was to provide a comprehensive idea about the potential role of EVs in facilitating non-lytic intercellular spread of an array of non-enveloped and enveloped viruses. The collective research performed so far has provided the fuel for further elucidating the complex interplay between viruses and EVs, eventually allowing us to better respond to newly emerging viral diseases in the future.

## Data Availability

No new data were created or analyzed in this study. Data sharing is not applicable to this article.
